# Incidence of Extrusion Following Type I Polypropylene Mesh “Kit” Repairs in the Correction of Pelvic Organ Prolapse

**DOI:** 10.1155/2012/354897

**Published:** 2011-12-10

**Authors:** James C. Lukban, Roger D. Beyer, Robert D. Moore

**Affiliations:** ^1^Division of Urogynecology, Department of Obstetrics and Gynecology, Eastern Virginia Medical School, 825 Fairfax Avenue, Suite 310, Norfolk, VA 23507, USA; ^2^Women's Health Care Specialists, PC, 7110 Stadium Drive, Kalamazoo, MI 49009, USA; ^3^Atlanta Urogynecology Associates, 3400 Old Milton Parkway, Building C, Suite 330, Alpharetta, GA 30005, USA

## Abstract

*Introduction and Hypothesis*. We sought to determine the mesh extrusion (vaginal exposure) rates and subject outcomes following IntePro (Type I polypropylene) mesh “kit” repairs for vaginal prolapse. *Methods*. Data were pooled from two prospective multicenter studies evaluating the safety and efficacy of the Perigee and Apogee (American Medical Systems, Minnetonka, Minn, USA) to treat anterior and posterior/apical prolapses, respectively. Extrusions involving the anterior compartment (AC) or posterior compartment/apex (PC/A) were recorded. *Results*. Two hundred sixty women underwent mesh placement, with a total of 368 mesh units inserted (173 in the AC and 195 in the PC/A). Extrusions were noted in 13 (7.5%) of AC implants and 27 (13.8%) of PC/A implants through 12 months. No difference was seen between those with and without extrusion in regard to anatomic cure, postoperative painor quality of life at 1 year. *Conclusions*. Extrusion had no apparent effect on short-term outcomes. Given the unknown long-term sequellae of vaginal mesh exposure, a thorough assessment of risks and benefits of transvaginal mesh placement should be considered at the time of preoperative planning.

## 1. Introduction

Pelvic organ prolapse (POP) represents an attenuation or disruption of the vaginal muscularis comprising the pubocervical “fascia” anteriorly or rectovaginal “fascia” posteriorly, manifesting as anterior or posterior vaginal wall prolapse, respectively. Additionally, a weak or torn cardinal-uterosacral ligament complex may lead to apical or uterine descent. Traditional transvaginal correction of vaginal wall defects through native tissue repair relies on the use of compromised muscular and connective tissue elements. Such repairs exhibit variable durability as evidenced by an overall reoperation rate approaching 30% [1]. The prospective success rates of traditional anterior colporrhaphy range from 30% to 81% at a mean followup of 1 to 2 years [2–6].

In the correction of posterior vaginal wall prolapse, traditional rectovaginal “fascial” plication and site-specific defect repairs have yielded retrospective success rates ranging from 76% to 82%, representing variable definitions of cure with followup from 6 to 42.5 months [7–9]. In an effort to enhance anatomic durability, surgeons have employed a number of biologic grafts or absorbable synthetic mesh to reinforce a traditional repair or replace disrupted or deficient vaginal muscularis. Outcomes from a number of randomized controlled trials (RCTs) evaluating the efficacy of such products in both anterior and posterior compartments are variable [3–6, 10].

The use of nonabsorbable synthetic mesh has been the most recent evolution in vaginal reconstructive surgery, with data from the general surgery literature confirming anatomic durability following abdominal wall hernia repairs employing permanent material [11]. Initial prospective data from Julian and Grody showed significant benefit from nonabsorbable mesh in the anterior compartment. Subjects receiving Marlex for reinforcement over plication/paravaginal repair had less recurrence than those receiving plication/paravaginal repair alone, reporting success rates of 100% and 67%, respectively (followup of 2 years) [12]. More recent RCTs on the use of nonabsorbable mesh in the anterior compartment have also shown significantly greater anatomic durability following the addition of polypropylene mesh to native tissue plication [4, 13, 14].

Nonabsorbable synthetic mesh in the posterior compartment was evaluated prospectively by Rutman et al., reporting a 98% success rate in the treatment of rectocele [15]. In an RCT by Withagen et al., the use of nonabsorbable synthetic mesh for posterior repair resulted in better anatomic durability versus native tissue, with 12-month success rates of 95.9% and 75.5%, respectively, [14].

The most commonly described nonabsorbable synthetic mesh in both the gynecologic and urologic literature is Type I polypropylene, which possesses the mechanical properties of durability, elasticity, and resistance [16] in addition to the in vivo characteristics of good tissue integration with minimal inflammatory response [17]. Placing polypropylene mesh in the vagina, however, is associated with an inherent risk of extrusion on the order of 2.3% to 25% [12, 18–22] from prospective data for the anterior wall and 2% to 12% [15, 23] for the posterior wall. Mesh “kits” have been available since 2004, offering the theoretical benefit over free mesh in enabling placement of synthetic material without significant dissection, reducing operative morbidity allowing for insertion without tension, and preserving normal visceral function. Early retrospective data on 77 subjects receiving Perigee showed an average blood loss of 77 cc with no postoperative reports of urinary retention [24].

Our objective was to determine the rate of extrusion in subjects receiving Type I polypropylene mesh in the repair of pelvic organ prolapse employing Perigee (to treat anterior vaginal prolapse) and/or Apogee (to treat posterior and/or apical vaginal prolapse).

## 2. Materials and Methods

### 2.1. Study Design

Data were collected from two prospective multicenter clinical studies evaluating the safety and efficacy of two IntePro (Type I, large pore, polypropylene mesh) “kits.” The PERIGEE Synthetic Study enrolled subjects with anterior vaginal prolapse utilizing the Perigee System (American Medical Systems, Minnetonka, Minn, USA), and the PROPEL Study (Phase I) enrolled subjects with posterior vaginal prolapse and/or apical descent for which the Apogee System was employed. Concomitant repairs of nonstudy vaginal wall compartments with IntePro were permitted in each trial allowing for evaluable data on both devices from either study. Each had similar protocols, inclusion/exclusion criteria, and data collection such that pooling of data for purposes of examining mesh complications and outcomes was deemed appropriate.

All sites received institutional review board approval prior to enrollment. Each investigator was required to have performed a minimum of five Perigee and five Apogee implants prior to participation. Subjects were required to have a diagnosis of prolapse ≥ Stage II by the pelvic organ prolapse quantification system (POP-Q) [25] in the compartment undergoing correction and were excluded with any of the following conditions: prior graft-augmented repair; systemic or local conditions that would preclude surgery or affect healing such as coagulation disorders, infection, compromised immune response, vaginal bleeding, erosion, tissue necrosis, or uncontrolled diabetes mellitus; restricted leg motion (inability to conform to the lithotomy position).

Data were collected at baseline, procedure, 3 months, 6 months, and 12 months. Physical examination of the surgical site was conducted at each visit and mesh extrusions were recorded as to compartment, days to onset, intervention for resolution, and location of vaginal wall exposure (PROPEL Study only). POP-Q measurements were completed by the same trained individual at each institution at baseline, 6 months, and 12 months to preserve comparative validity. Pelvic pain scores (Wong-Baker FACES Pain Rating Scale ranging from 0 for “no pain” to 10 for “worst pain”) [26] were recorded at baseline, 6 weeks, and 3 months. Validated quality of life (QoL) questionnaires including the Pelvic Floor Distress Inventory (PFDI), Pelvic Floor Impact Questionnaire—Short Form 7 (PFIQ-7), and Pelvic Organ Prolapse Urinary Incontinence Sexual Function Questionnaire (PISQ-12) were completed by subjects at baseline and at 12 months [27–29]. A Patient Satisfaction Survey was also administered at 6 and 12 months to subjects in the PROPEL Trial.

### 2.2. Surgical Procedures

Each subject underwent transvaginal placement of IntePro (mesh density 50 grams/M^2^) by means of bilateral double transobturator trocars for Perigee and bilateral transgluteal trocars for Apogee. Mesh was anchored to the pelvic sidewall via self-fixing appendages (4 for Perigee and 2 for Apogee) and customized to the subject's anatomy and repair requirements by means of trimming. The appendages are constructed of a polypropylene monofilament that is precut to 1.1 cm in width ×23 cm in length and have properties that allow for tissue anchoring and ingrowth. A single polypropylene tensioning suture is prethreaded through the length of each appendage to allow for tensioning after placement. Each mesh system is intended to remain in the body as a permanent implant and is not absorbed or degraded by the action of ingrowth or tissue enzymes.

Preoperatively, patients received intravenous cephalosporin or quinolone within an hour of surgery. Operative techniques for Perigee and Apogee were as previously described [24, 30]. In general, a vertical midline incision ≤5 centimeters in length was employed in both the anterior and posterior compartments. A full-thickness dissection was achieved following infiltration with local anesthetic for both hemostasis and dissection of planes. Little or no trimming of the vaginal muscularis was performed, and closure was with delayed absorbable suture employing a technique at the discretion of the surgeon. Additional reconstructive procedures were performed as indicated with the exception of concomitant repairs in the same vaginal segment. Vaginal packing was placed and removed within 12–24 hours.

### 2.3. Analyses

Patients who received mesh were categorized into four groups: (1) subjects with mesh in the anterior compartment who experienced extrusion along the anterior vaginal wall; (2) subjects with mesh in the anterior compartment who did not experience extrusion; (3) subjects with mesh in the posterior compartment/apex who experienced extrusion along the posterior vaginal wall/apex; (4) subjects with mesh in the posterior compartment/apex who did not experience extrusion.

Anatomic cure was defined as POP-Q ≤ Stage I for each vaginal segment. Cure rates were compared between the extrusion and nonextrusion groups for each compartment. Within-group and between-group comparisons were carried out for mean Wong Baker FACES pain scale, PFDI, PFIQ-7, and PISQ-12 scores.

Continuous data were summarized as mean ± SD. Count and percent were reported for categorical data. Wilcoxon rank-sum test was used for between group (unpaired) comparisons, and the paired *t*-test or signed rank test was used for within group (paired) comparisons. Chi-square or Fisher exact tests were employed to compare categorical outcomes between groups, and McNemar test was employed to compare categorical outcomes within groups. Univariate analysis was used to assess the effect of potential risk factors for extrusion. A *P* value <0.05 was considered statistically significant. Data were analyzed using SAS version 9.2 (Cary, NC, USA).

## 3. Results

Two hundred sixty women underwent IntePro placement (173 in the AC and 195 in the PC/A) during participation in either of the two studies at a total of 19 academic and community urogynecologic, urologic, or gynecologic practices in the United States. All subjects have passed beyond the 12-month post procedure followup time point. Mean followup was 10.9 ± 3.1 and 10.9 ± 3.0 months for the AC and PC/A groups, respectively, as some subjects were lost to followup prior to one-year. Thirteen of 173 (7.5%) subjects who had Perigee experienced mesh extrusion along the anterior wall. Twenty-seven of 195 (13.8%) subjects receiving Apogee experienced mesh extrusion along the posterior or apical portion of the vagina. One subject who received Apogee (0.5%) experienced an erosion of IntePro into the rectum detected 401 days after implant by routine fecal occult blood testing. This event was successfully treated in the operating room by transanal trimming of exposed mesh (3 mm) followed by a two-layer closure. Following repair, the subject maintained anatomic success and experienced no further sequelae. There were no other erosions of mesh into the bladder or rectum reported in either study.

Subjects with extrusions exhibited similar baseline characteristics to those without, except for prior hysterectomy in the AC group. Age, moderate to severe vaginal atrophy determined by vaginal pH, or history of prior failed repairs in the same compartment as possible covariates were not significant risk factors for extrusion for either AC or PC/A mesh placement (Table 1).

Vaginal exposure of mesh occurred at a median of 95 days and 93 days after surgery for the AC and PC/A, respectively, (Table 2). Twenty-three percent (3/13) of AC and 48.1% (13/27) of PC/A extrusions were treated noninvasively through application of vaginal estrogen cream, antibiotics, and/or trimming of exposed mesh in an office setting. All others were treated by trimming of mesh and repair of the epithelial defect in the operating room. No extrusion or erosion required removal of the entire mesh system; however, one subject underwent removal of the central portion. Data on the location of extrusions (midline, apical, or distal) were available for subjects participating in the PROPEL Study only (Table 3). Eight subjects (61.5%) with a PC/A extrusion and two subjects (100%) with an AC extrusion exhibited a midline exposure. Eight percent (1/13) of those with extrusion of the PC/A had an apical extrusion.

Baseline and followup anatomic evaluations, perioperative pain scores, and QoL analyses are presented in Table 4. The percentage overall of Perigee (AC) patients with anatomic cure at 12 months was 84.2% (123/146). The percentage overall of Apogee (PC/A) subjects with anatomic cure as measured by apical staging was 92.5% (37/40), and by posterior wall staging was 95.6% (152/159) at 12 months. No difference in anatomic success was seen between extrusion versus nonextrusion patients.

Mean pain scores were not significantly different between subjects with and without vaginal mesh extrusion in the AC or PC/A at baseline and at 12 months. QoL analyses showed similar improvement (from baseline to followup at 12 months) in extrusion versus nonextrusion subjects in each compartment. Dyspareunia (subject self-report from PISQ-12, number 5) in the AC and PC/A groups was neither significantly different at 12 months compared to baseline, nor between groups at 12 months.

Patient satisfaction was recorded in the PROPEL Study and revealed 96.4% (106/110) to report “some” or “a lot” of improvement, 94.5% (104/110) who were “moderately,” “very,” or “extremely” satisfied, and 100% who would “recommend this procedure to a friend suffering from prolapse.”

## 4. Discussion

Our extrusion rate of 7.5% in the AC is higher than that reported by Nguyen and Burchette, who identified 2 of 37 subjects (5%) to exhibit vaginal exposure at 1 year as part of a randomized controlled study comparing Perigee with anterior repair to tissue plication alone [13]. Both subjects were treated conservatively. Moore and Miklos retrospectively reported a 6.5% extrusion rate in 77 subjects undergoing Perigee at a mean followup of 18.2 months [24]. Four extrusions out of 72 subjects receiving Perigee (5.6%) were reported by Gauruder-Burmester et al., with all requiring revision in an unknown setting [30]. Two out of 32 subjects (6.3%) receiving Perigee and 3 out of 30 (10%) implanted with Apogee exhibited extrusion in a retrospective analysis of 70 subjects treated with IntePro, with all cases unresponsive to conservative therapy [31]. This 10% rate of posterior vaginal wall extrusion is less than the 13.8% found in our studies.

Vaginal extrusion in our pooled samples occurred at a median onset of 95 days (range 34–426) and 93 days (range 3–418) for the AC and PC/A, respectively. The cases of delayed exposure are consistent with data from Boulanger et al., who reported a mean interval of 26.6 months between vaginal mesh implant and removal in 16 patients (of whom 62% exhibited symptomatic extrusion) [17]. In an effort to determine etiology, bacterial analysis of each of the 16 explants was performed, with culture most commonly identifying *Streptococcus* species. As none of the colony counts were found to exceed 10^4^, the role of bacterial contamination in regard to mesh complications was deemed unclear by the authors. Similar data were reported by Petros et al. in an early retropubic sling feasibility study, who identified bacterial concentrations of <10^5^  in 8 explanted synthetic tapes having been placed with free vaginal ends in a canine model for up to 19 weeks [32].

No correlations were identified in our study between age, baseline vaginal pH, or history of prior failed repairs in the same compartment. The absence of a correlation with age differs from findings by Deffieux et al. who reported an increased risk of polypropylene mesh extrusion with increased age (>70 years) [33]. Our choice of vaginal pH as an indicator of local estrogenization (presence of vaginal atrophy) was chosen as a covariate, as hormone replacement therapy may vary in terms of route of delivery, dose prescribed, length of administration, and subject compliance. In our studies, the lack of correlation between estrogen status (moderate to severe vaginal atrophy) at baseline and extrusion rate is consistent with a previously unpublished study [34]. Moore et al. prospectively examined 98 women receiving Perigee with IntePro with baseline vaginal mucosal maturation cytological indices. Six (6.1%) subjects exhibited vaginal extrusion of mesh of which only 1 had a low estrogen effect (severe vaginal atrophy) and the rest an adequately estrogenized vaginal epithelium.

The finding of prior hysterectomy as a risk factor for extrusion in subjects treated with Perigee is surprising, given that no such correlation was found between PC/A mesh placement employing Apogee in which contact between polypropylene and the apical scar may have been more common. Unpublished data has been presented on a subgroup of patients from the PROPEL Study in which a similar apical extrusion rate was found in those with (2.8%) and without (1.0%) hysterectomy at the time of Apogee with IntePro placement in the PC/A [35]. As reported by Collinet et al., hysterectomy with an inverted “T” colpotomy was an independent risk factor for extrusion in a sample of subjects receiving a Prolene transvaginal mesh [36].

In the PROPEL Study, we examined specific sites of mesh extrusion to determine where along the vaginal wall subjects were most prone to exposure, determining that the majority of exposures occurred along the midline. This finding may be consistent with wound separation associated with mesh contracture and/or hematoma [37].

Extrusions were treated conservatively (vaginal estrogen cream, antibiotics, and/or trimming in the office) in approximately 25–50% of cases, and those returning to the operating room (5.8% for Apogee and 7.2% for Perigee overall) for excision were treated by mesh trimming and closure of the wound without major revision or complete system explant.

An additional, yet unproven, risk factor for vaginal exposure of mesh may be that of material density, as the lighter weight mesh (25.5 grams/M^2^) comprising the second-generation elevate has yielded lower extrusion rates compared to those seen in our studies. In unpublished prospective data, anterior elevate (American Medical Systems, Minnetonka, Minn, USA) was found to exhibit a mesh exposure rate of 5.6% at 1 year, while posterior elevate (American Medical Systems, Minnetonka, Minn, USA) exhibited rates of 6.5% and 7.9% at 1 and 2 years, respectively, [38–40].

A single case of erosion into the rectum was reported in our studies, representing 1/369 or 0.3% of mesh units placed. Such events also remain rare in the literature. Abdel-Fattah and Ramsay reported one erosion into the bladder following an anterior Gynecare mesh out of a 146 subjects receiving polypropylene in the AC (representing an incidence of 0.7%) with no erosions reported in 149 subjects implanted in the PC [31].

Anatomic success was high at one year for all patients in our analysis with no difference in anatomic durability in those with and without extrusion. In a study by Bellon et al., polypropylene showed no difference in tensile strength compared with controls upon inoculation with *Staphylococcus epidermidis* or *Staphylococcus aureus* 30 days after implant into the abdominal wall of a rabbit model [41].

Mean postoperative pain scores were similar in both groups, and QoL showed similar improvements from baseline to followup for extrusion versus nonextrusion subjects in each compartment. Patient satisfaction overall as recorded for PROPEL subjects was high.

The strength of this study is the prospective enrollment and collection of data from a large number of subjects with a minimum followup of 12 months. Validated instruments for measurement of anatomic success, and QoL provided uniformity in data interpretation. Presentation of data on the incidence of extrusion in the context of anatomic durability and outcomes allows for a measure of confidence in risk-benefit evaluations in regard to the use of mesh “kits.”

Study limitations include the large number of surgeons involved in device implantation, perhaps representing a range of techniques in regard to wound closure, especially, with the potential for variations in this regard as a contributor to the incidence and site of extrusion in some subjects. Since Perigee and Apogee used in this trial were among the first kits available on the market, enrollment early in the product life cycle versus later enrollment may affect success and complication rates as opposed to rates seen today in the clinical setting. Patients enrolled in the PROPEL Study represented a sample implanted at a time more remote from Apogee market introduction, and in unpublished data, exhibited an extrusion rate of 8.4% at a mean of 11 months [42]. This lower incidence of vaginal exposure as compared to the number generated from our pooled data from two sequential studies reasonably represents a “learning curve.” As we were without a predetermined sample size, the absence of a significant difference in observed outcomes (anatomic durability, postoperative pain, and QoL) between those with and without extrusion may represent and underpowered scenario.

Based on our finding of a preponderance of midline extrusions, it would be reasonable to suggest a thick dissection with minimal to no trimming of the vagina, in addition to a meticulous closure. Additionally, presumptive measures to reduce bacterial colonization such as appropriate perioperative antibiotics, thorough perineal preparation, draping of the anal verge, and generous intraoperative irrigation would be appropriate. Excellent hemostasis to reduce hematoma formation with vaginal packing could also be beneficial. Frequent and prolonged followup would be prudent to allow for extrusion detection and intervention prior to the progression of mesh exposure to a point that may require extensive revision.

## 5. Conclusion

Vaginal extrusion of mesh appears to have no impact on anatomic durability, postoperative pain or QoL at 1 year. Given the unknown long-term sequellae of vaginal mesh exposure, a thorough assessment of risks and benefits of transvaginal mesh placement should be thoughtfully considered.

## Figures and Tables

**Table 1:** Subject demographics.

	AC mesh, *N* = 173			PC/A mesh, *N* = 195		
Baseline characteristic	Extrusion *N* = 13	No extrusion *N* = 160	*P* value	Extrusion *N* = 27	No extrusion *N* = 168	*P* value
Age*	58.6 ± 10.4,	60.8 ± 12.8,	0.48^W^	56.3 ± 15.3,	58.7 ± 12.2,	0.37^W^
BMI	25.4 ± 3.7,	27.8 ± 5.9,	0.21^W^	28.2 ± 7.9,	28.6 ± 5.6,	0.42^W^
Parity	3 ± 2,	3 ± 2,	0.53^W^	3 ± 1,	3 ± 2,	0.28^W^
Prior failed prolapse procedure: Cystocele*	4 (30.8%)	25 (15.6%)	0.24^F^	—	—	—
Prior failed prolapse procedure: Rectocele*	—	—	—	3 (11.1%)	9 (5.4%)	0.22^F^
Prior failed prolapse procedure: apical*	—	—	—	2 (7.4%)	7 (4.2%)	0.36^F^
Prior hysterectomy*	12 (92.3%)	74 (46.3%)	0.00^C^	13 (48.1%)	84 (50.0%)	0.86^C^
Postmenopausal	13 (100%)	125 (78.1%)	0.07^F^	20 (74.1%)	126 (75.0%)	0.92^C^
Estrogen therapy at baseline	9 (69.2%)	72 (45.0%)	0.09^C^	15 (55.6%)	87 (51.8%)	0.72^C^
Diabetic	1 (7.7%)	13 (8.1%)	1.00^F^	3 (11.1%)	17 (10.1%)	1.00^F^
Moderate to severe vaginal atrophy at baseline (based on pH)*	3 (23.1%)	39 (24.4%)	1.00^F^	6 (22.2%)	33 (19.6%)	0.77^C^

*Covariates assessed for the risk of an extrusion.

^W^Wilcoxon rank sum test; ^C^chi-square test; ^F^Fisher exact test.

AC: anterior compartment; PC/A: posterior compartment/apex; BMI: body mass index.

**Table 2:** Extrusion incidence and treatment.

Extrusion	AC mesh + extrusion	PC/A mesh + extrusion
*N*	13/173 (7.5%)	27/195 (13.8%)
Days to onset		

Mean	155	123
Median	95	93
Min	34	3
Max	426	418
Mesh trimmed in OR	10/13 (76.9%)	14/27 (51.9%)
Noninvasive Treatment*	3/13 (23.1%)	13/27 (48.1%)

*Noninvasive treatment consisted of application of vaginal estrogen cream, antibiotics, and/or trimming of exposed mesh in the office.

AC: anterior compartment; PC/A: posterior compartment/apex.

**Table 3:** Extrusion location (PROPEL Study).

Location of extrusion	AC Mesh + extrusion *N* = 2/59	PC/A Mesh + extrusion *N* = 13/141
Midline	2/2 (100%)	8/13 (61.5%)
Apical	—	1/13 (7.7%)
Distal	—	4/13 (30.8%)

AC: anterior compartment; PC/A: posterior compartment/apex.

**Table 4:** Outcome variables for subjects with and without extrusion (AC or PC/A).

Variable^1^	Baseline extrusion	Baseline nonextrusion	12-Month extrusion	12-Month nonextrusion	*P* value (within group)^2^	*P* value (between groups)^3^
Anterior compartment						

POPQ	13/13	160/160	1/12	22/134	NA	NA
Anterior stage ≥ II	(100.0%)	(100.0%)	(8.3%)	(16.4%)	NA	0.692

Wong-Baker	1.8 ± 2.8	1.6 ± 2.0	1.3 ± 2.4	0.5 ± 1.1	0.781	0.685
Pain score^ 4^	(*n* = 13)	(*n* = 160)	(*n* = 13)	(*n* = 151)	<.001	0.228

PFDI (symptoms)	44.6 ± 24.6	35.7 ± 26.2	16.7 ± 20.4	14.4 ± 20.4	0.024	0.243
Anterior POPDI	(*n* = 13)	(*n* = 160)	(*n* = 12)	(*n* = 135)	<.001	0.715

PFDI (symptoms)	130.2 ± 58.2	101.4 ± 55.6	40.0 ± 43.3	32.3 ± 39.4	<.001	0.075
UDI	(*n* = 13)	(*n* = 160)	(*n* = 12)	(*n* = 135)	<.001	0.523

PFIQ-7 (life impact)	31.1 ± 37.0	16.6 ± 24.0	12.7 ± 26.9	3.6 ± 12.4	0.008	0.186
POPIQ	(*n* = 13)	(*n* = 159)	(*n* = 12)	(*n* = 134)	<.001	0.267

PFIQ-7 (life impact)	42.9 ± 33.4	32.8 ± 27.4	19.8 ± 26.6	8.1 ± 15.9	0.061	0.211
UIQ	(*n* = 13)	(*n* = 160)	(*n* = 12)	(*n* = 134)	<.001	0.158

PISQ-12	31.1 ± 7.7	32.0 ± 7.1	34.1 ± 8.7	37.5 ± 5.9	0.151	0.693
(If sexually active)	(*n* = 10)	(*n* = 84)	(*n* = 8)	(*n* = 67)	<.001	0.149

Dyspareunia	5/10	36/83	5/8	20/67	1.000	0.745
(From PISQ-12, number 5)	(50.0%)	(43.4%)	(62.5%)	(29.9%)	0.491	0.108

Posterior compartment/Apex						

POPQ	5/27	42/168	1/23	4/141	0.180	0.629
Apical stage ≥ II	(18.5%)	(25.0%)	(4.3%)	(2.8%)	<.001	0.535

POPQ	27/27	163/168	0/23	7/141	NA	1.000
Posterior stage ≥ II	(100.0%)	(97.0%)	(0.0%)	(5.0%)	<.001	0.595

Wong-Baker	1.1 ± 1.9	2.0 ± 2.5	0.7 ± 0.9	0.5 ± 1.0	0.367	0.094
Pain score^ 4^	(*n* = 27)	(*n* = 167)	(*n* = 26)	(*n* = 162)	<.001	0.409

PFDI (symptoms)	48.5 ± 38.3	47.2 ± 36.0	27.2 ± 35.4	19.5 ± 27.9	0.001	0.863
POPDI posterior	(*n* = 27)	(*n* = 167)	(*n* = 23)	(*n* = 141)	<.001	0.242

PFDI (symptoms)	133.2 ± 89.6	127.7 ± 85.5	68.4 ± 79.5	50.2 ± 60.6	<.001	0.759
CRADI	(*n* = 27)	(*n* = 167)	(*n* = 23)	(*n* = 141)	<.001	0.204

PFIQ-7 (life impact)	21.7 ± 27.5	19.1 ± 25.8	2.9 ± 8.5	4.6 ± 14.2	<.001	0.637
POPIQ	(*n* = 27)	(*n* = 165)	(*n* = 23)	(*n* = 140)	<.001	0.432

PFIQ-7 (life impact)	18.3 ± 26.6	22.9 ± 27.2	10.1 ± 18.3	4.8 ± 13.9	0.093	0.415
CRAIQ	(*n* = 27)	(*n* = 165)	(*n* = 23)	(*n* = 140)	<.001	0.105

PISQ-12	33.4 ± 6.4	32.3 ± 7.4	38.6 ± 4.9	36.9 ± 6.2	0.046	0.592
(If sexually active)	(*n* = 15)	(*n* = 104)	(*n* = 15)	(*n* = 83)	<.001	0.323

Dyspareunia	7/15	43/105	3/15	28/83	0.083	0.782
(From PISQ-12, number 5)	(46.7%)	(41.0%)	(20.0%)	(33.7%)	0.450	0.376

^1^Values are presented as *N* (%) or Mean ± SD; NA: Not Available.

^2^ *P* value paired comparison between baseline and 12 Months for the extrusion group and nonextrusion group.

^3^ *P* value unpaired comparison between extrusion group and nonextrusion group for the baseline time-point and the 12-month time-point.

^4^Data at 3-month followup (Wong-Baker FACES pain scores were not collected at 12-month).
